# Pension exposure and health: Evidence from a longitudinal study in South Africa

**DOI:** 10.1016/j.jeoa.2022.100411

**Published:** 2022-10

**Authors:** Carlos Riumallo Herl, Chodziwadziwa Kabudula, Kathleen Kahn, Stephen Tollman, David Canning

**Affiliations:** a Erasmus School of Economics, Erasmus University Rotterdam, The Netherlands; b Tinbergen Institute, The Netherlands; c MRC/Wits Rural Public Health and Health Transitions Research Unit, School of Public Health, Faculty of Health Sciences, University of the Witwatersrand, South Africa; d Global Health and Population, Harvard T.H. Chan School of Public Health; e INDEPTH Network, Accra, Ghana

**Keywords:** Pensions, Ageing, South Africa, Health, Self-reported disabilities

## Abstract

Social protection schemes have been expanding around the world with the objective of protecting older persons during retirement. While theoretically they have been seen as tools to improve individual wellbeing, there are few studies that evaluate whether social pensions can improve health. In this study, we exploit the change in eligibility criteria for the South African Old Age grant to estimate the association between pension exposure eligibility and health of older persons. For this, we use data from the Health and Aging in Africa: A longitudinal Study of an INDEPTH Community in South Africa (HAALSI) and model pension exposure in terms of its cumulative effect. Our results show that pension exposure is associated with better health as measured by a set of health indices. Disentangling these effects, we find that pension exposure is most likely to improve health through the delayed onset of physical disabilities in the elderly population. Our study highlights the relevance of social protection schemes as a mechanism to protect older persons physical health.

## Introduction

Social protection schemes for older persons have been expanding in many low and middle–income countries (Barrientos and Hulme, 2009, Barrientos, 2013). While the main objectives of these programs are to protect the elderly financially in retirement or to contribute financially to households in poorer communities, it is likely that they have a broader effect on individual wellbeing. This possibility has led to an increased interest in evaluating the role of social protection schemes in promoting healthy ageing within low and middle–income countries. The evaluation of these policies is further motivated by the fact that by 2050, more than one in five individuals will be over the age of 60, and 80 % of older individuals will be living in low– and middle–income countries (World Health Organization, 2015). Consequently, all countries are attempting to design policies that not only protect individuals and contribute financially to households in poorer communities but also protect the health of the elderly (Faulbaum and Vargas. , 2021, Niño-Zarazúa, 2012).

It is intuitive to think that social pension schemes can have a positive influence on older persons' health. On one hand, many studies in the health economics and public health literature show that generally greater income leads to better health behaviours and improved access to health care (e.g. Finkelstein, 2012, Laaksonen and Ritva Prättälä; Ville Helasoja; Antti Uutela and Eero Lahelma. , 2003, Phelan and C; Bruce G Link and Parisa Tehranifar. , 2010, Riumallo-Herl and Aguila, 2019). On the other hand, there is also evidence that pension income can lead to increased consumption of non–medical products that can enhance health (Peter Lloyd-Sherlock and Sutapa Agrawal, 2014).

Despite the extensive evidence highlighting the potential mechanisms through which social pensions can lead to better health, the effect of pension income on health remains understudied. More importantly, some of the current findings in the literature suggest that there is no effect from social pensions on health. A series of studies from sub–Saharan Africa suggest that either there is no effect of social pensions on health, or that the benefits are short–lived (Lloyd-Sherlock and Agrawal, 2014, Lloyd-Sherlock et al., 2020, Lloyd-Sherlock, 2012, Lloyd-Sherlock, 2012). This contradicts evidence from other studies that find pensions lead to improved health in North America or Asia (Aguila et al., 2015, Águila et al., 2018, Pak, 2021). An important challenge in some of these studies has been the limited availability of health data to examine the question.

In this study, we explored the effect of pension exposure eligibility on health using data from the Health and Aging in Africa: A longitudinal Study of an INDEPTH Community in South Africa (HAALSI). This survey collected comprehensive economic and health data from 2014 to 2019 on an ageing population in Agincourt, rural South Africa. We explored the role of pension exposure eligibility by calculating how long an individual has been eligible for the old age grant thus providing an estimate of the cumulative effect of old age pensions. We measure eligibility as it correlates strongly with receipt but avoids the endogenous behaviour of pension uptake. In terms of health, our main outcomes are three health indices based on previous ageing surveys (Debpuur et al., 2010, Gómez-Olivé, 2010, Hirve, 2010, Kyobutungi et al., 2010, Meijer et al., 2011, Mwanyangala, 2010, Ng, 2010, Poterba, 2010, Razzaque, 2010, Riumallo-Herl et al., 2019, Minh, 2010). We found that pension exposure eligibility is positively associated with better health, but the patterns depend on the health outcome. More specifically, we found that being eligible for the old age grant is associated with better general health, particularly influencing aspects of self–reported disability. This is consistent with the current evidence suggesting that pensions can improve food availability which in turn may be responsible for delaying the onset of physical disabilities (Lloyd-Sherlock and Agrawal, 2014, Robinson, 2018).

Our study contributes to a growing literature in economics of ageing that explores the impact of pensions on health in low– and middle–income countries in several ways. First, our study shows that in contrast to previous studies, the effect of pensions on health can be long–lasting rather than temporary (Enid Schatz, Xavier Gómez-Olivé, Margaret Ralston, Jane Menken and Stephen Tollman, 2012). Second, many earlier studies focus on the effect of being eligible but dismiss the possibility that pension exposure can have a cumulative effect. In our models we include exposure to evaluate the cumulative effect of pension exposure. From this, we find differential impacts of pension exposure on our health outcomes. Third, our findings show that pension exposure can influence health in Sub–Saharan Africa and more importantly highlight that such effect may be occurring through specific health domains. Finally, our study provides supporting evidence for other studies in low– and middle– income countries where a positive impact of pensions has been found on health (Emma Aguila, Arie Kapteyn and James P Smith, 2015, Emma Águila, Mariana López-Ortega and Luis Miguel Gutiérrez Robledo, 2018, Peter Lloyd‐Sherlock, Nadia Minicuci, John Beard and Somnath Chatterji, 2012, Tae-Young Pak, 2021).

Our results showcase the potential role that social protection has in encouraging healthy ageing in developing countries. As populations continue to age, policy makers should consider the expansion of social protection schemes for the elderly not only as a financial protection tool, but also a mechanism through which to improve individual wellbeing.

## Methods

### Institutional setting

South Africa currently hosts one of the largest and most generous old age pension schemes in developing countries (Stephen Devereux, 2007; Stewart and Yermo, 2009). The first laws concerning old age pensions were established in 1928 when a means–tested social pension was implemented to cover white male workers and women of mixed race without access to occupational pensions (ILO, 2016). Throughout most of the 20th century, the old–age social pension would remain exclusively available for these populations. Only a small fraction of the coloured population had access, and even then, the amounts received would be substantially lower than white recipients (Seekings and Nattrass, 2008).

**Fig. 1:**
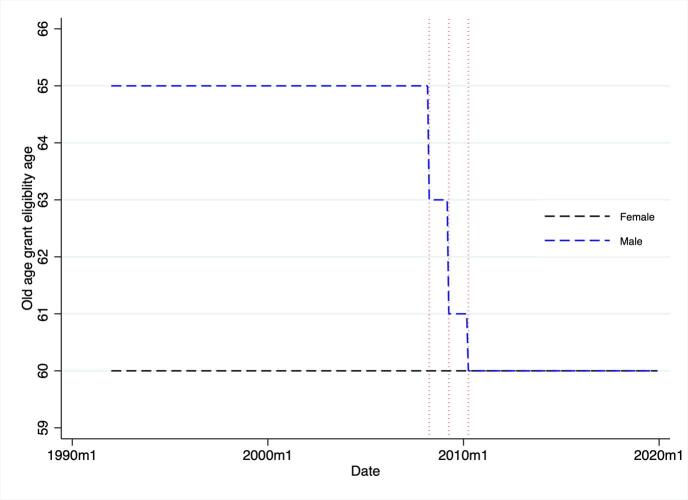
South African Old Age State Grant Eligiblity: Age Over Time.

The demise of apartheid brought considerable changes to the old–age social pension scheme. In 1992, the means–tested grant was expanded to all population groups and the amounts received were equalised for every-one. Nevertheless, the age eligibility criteria were maintained at 65 years for men and 60 for women. In 2008, an amendment was legislated by government that modified the age eligibility criteria for the old age state grant (Republic of South Africa, 2018). In particular, the age eligibility for men was reduced from 65 to 60 years between 2008 and 2010. Fig. 1 shows the age eligibility criteria for men and women between 1990 and 2020, highlighting the step–wise reduction in age of male eligibility that resulted from the reform.

Since the 2008 reform, all South African citizens, permanent residents, or designated refugees living in South Africa are eligible for the old age grant if they are older than 60 years (Republic of South Africa, 2018). The old age grant remains a means–tested social pension and therefore certain annual income and asset thresholds are used to determine eligibility. Individuals may receive the grant if they are not receiving any other grant; and if their income and assets are lower than ZAR 86,280 (∼USD 5,400) per year and ZAR 1.2 million (∼USD 79,000) respectively if they are single, or ZAR 172,560 (∼USD 11,300) and ZAR 2.4 million (∼USD 158,000) respectively if they are married (South African Government, 2022). To apply for the old age grant, eligible individuals must first go to the nearest South African Social Security Agency (SASSA) office with their identification and documentation.

After a review period, individuals awarded the old–age grant are paid from the date of application. Currently the benefit amounts are ZAR 1,890 (∼USD 120) per month for those aged between 60 and 75, and R1,910 (∼USD 125) per month for those older than 75 (Social Security Administration, 2019). The South Africa old age grant program is considered one of the most generous nowadays, not only because the monthly amounts represent approximately 50 % of the minimum monthly working wage, but also because it covers a large fraction of the population. In 2020, more than 90 % of those older than 60 years received the old–age grant (Statistics SA, 2021).

### Data

We used data from the baseline and first follow–up wave of HAALSI. The objective of this study is to inform health and social policy on ageing in rapidly transitioning South and sub-Saharan African settings with rigorous data collection from the population aged 40 and above representative of the rural Agincourt subdistrict which is underpinned by an established health and socio-demographic surveillance system (HDSS) covering some 120,000 people (Kathleen Kahn et al., 2012). The Agincourt HDSS is located in the northeast rural region of South Africa near the frontier with Mozambique and it covers a poor and rural African population. The first wave of data, collected in 2014 and 2015, consisted of a sample of 5,059 respondents which represented an 85.9 % response rate of the original population sample drawn from the HDSS. The second wave, conducted in Agincourt in 2018 and 2019, was able to obtain follow up 4,176 individuals - an 82.55 % follow up rate. Amongst those lost to follow up, the main reason was deaths (67 %) while the remaining individuals refused or were not found.

HAALSI is designed as a sister study of the US Health and Retirement Study (HRS) and seeks to obtain high quality economic and health information on an ageing population in South Africa. Hence, HAALSI collects detailed information on household economic, social network, and social conditions as well as detailed information on individual health including a combination of self–reported measures, anthropometry, cognitive measures, and biomarkers. This is supplemented by household and vital events data from the HDSS including on mortality, cause-of-death, and migrations. All information was collected via face–to–face interviews and a detailed description of the study protocol and cohort profile can be found in the literature (F Xavier Gómez-Olivé et al., 2016).

**Fig. 2:**
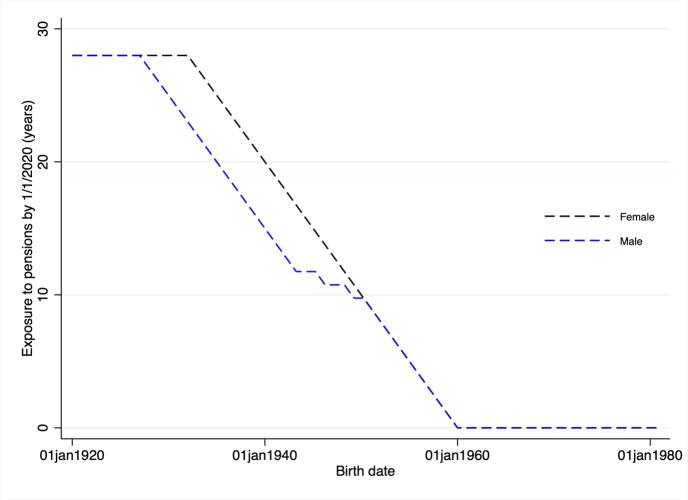
Old Age Grant Exposure in 2020 by Date of Birth.

In this study we estimated the association between pension exposure and individual health. To define pension exposure, we exploit the South African pension reforms from 1992 onwards to calculate for all respondents the number of years they have been eligible for the South African old age state grant. HAALSI covers individuals from the Agincourt HDSSS who are of African origin. Consequently, no individuals were eligible for social pensions before 1992 thus providing us with an initial exposure date for all individuals in the study sample. Fig. 2 presents the simulated number of years a person would have been exposed to the old age pension grant in 2020 by year of birth. Information on the reform timings and eligibility age is then used to construct the number of years potentially exposed to the old age state grant based on the person–specific date of birth and date of interview. Appendix Fig. 1 presents the distribution of the number of years individuals included in the HAALSI sample have been eligible for the old age state grant. Overall, a large fraction of the sample has not been eligible for the grant as they remain below the age–threshold of eligibility, while the number of eligible years ranges from 1 to approximately 30 for the remaining individuals in the sample. This figure also highlights the maximum number of years that an individual could have been eligible in line with the expansion of the old age pension system in 1992 to all qualifying individuals in South Africa.

To explore the effect of pension exposure on health, we constructed three summary health measures commonly used to evaluate the health of older persons. These measures were chosen as they have been routinely used in ageing studies, and particularly in studies using data from the INDEPTH network studies of demographic surveillance sites (Xavier Gómez-Olivé, Margaret Thorogood, Benjamin D Clark, Kathleen Kahn and Stephen M Tollman, 2010, Carlos Riumallo-Herl, David Canning and Chodziwadziwa Kabudula, 2019). All three measures use principal component analysis (PCA) to aggregate a set of health conditions using the weights for the first principal component. In our secondary analysis we explore the effect of pension exposure on the domains that make up the different indices to obtain a more nuanced understanding of the potential impact of pension exposure.

The first summary measure, which we call *health status,* follows a series of studies that have used the WHO Study on Health and Global Ageing (SAGE) data (Cornelius Debpuur, Paul Welaga, George Wak and Abraham Hodgson, 2010, Xavier Gómez-Olivé, Margaret Thorogood, Benjamin D Clark, Kathleen Kahn and Stephen M Tollman, 2010, Siddhivinayak Hirve, Sanjay Juvekar, Pallavi Lele and Dhiraj Agarwal, 2010, Catherine Kyobutungi, Thaddaeus Egondi and Alex Ezeh, 2010, MathewA Mwanyangala, Charles Mayombana, Honorathy Urassa, Jensen Charles, Chrizostom Mahutanga, Salim Abdullah and Rose Nathan, 2010, Nawi Ng, Mohammad Hakimi, Peter Byass, Siswanto Wilopo and Stig Wall, 2010, Abdur Razzaque, Lutfun Nahar, Masuma Akter Khanam and Peter Kim Streatfield, 2010, Hoang Van Minh, Peter Byass, Nguyen Thi Kim Chuc and Stig Wall, 2010). This measure relies on a set of self–reported measures that cover the following health domains: mobility, self–care, pain and discomfort, cognition, interpersonal activities, affect, and vision. We aggregated self–reported measures covering these domains to provide a comprehensive vision of an individual's personally reported level of health.

The second summary health measure, referred to as *functioning status* hereafter, is based on the WHO disability assessment schedule (T Bedirhan Üstün et al., 2010). This constructed summary measure provides a broad measure of the individual's physical and mental capacity. In contrast to the previous measure, the *functioning status* mainly includes measures on limitations in activities of daily living (ADLs), as well as self-reported measures of concentration and mental health. Finally, our third main outcome, referred to as *PVW Health Status,* follows the approach defined by Poterba, Venti and Wise (James M Poterba, Steven F Venti and David A Wise, 2010) to construct a health indicator that adds information on diagnosis and health care utilization to the self–reported domains available in the *health status* measure. The *PVW Health Status indicator* has been applied as a summary measure of elderly health (Kapteyn and Meijer, 2013; Meijer et al., 2011).

Each index is constructed independently for each wave such that higher values represent better health and lower values represent worse health. Appendix Fig. 2 shows the distribution of each health measure and Appendix Table 1 presents detailed information on the variables and weights used to construct each specific index. For interpretation purposes, our main analysis is conducted on the standardized health measures.

**Table 1:** Descriptive Statistics at Baseline, Agincourt, South Africa 2014/5.

	(1)	
	Mean	SD
Demographics:		
Age	62.39	(13.01)
Male	0.46	
No formal education	0.46	
Some primary education	0.34	
Some secondary education	0.11	
Secondary or higher education	0.09	
Never married	0.06	
Married or in partnership	0.51	
Separated or divorced	0.13	
Widowed	0.30	
Born in South Africa	0.70	
Working	0.16	
Total household consumption per capita	1584.27	(2134.58)
Exposure and pensions:		
Old age pension	0.50	(0.50)
Number of years eligible for pension	5.82	(7.48)
No exposure	0.44	
1–5 years	0.14	
6–10 years	0.16	
More than 10	0.25	
Health indices:		
Health status index	0.00	(1.00)
Functionality status index	−0.00	(1.00)
PVW Health status index	0.00	(1.00)
Health measures:		
Good or very good self-reported health	0.68	
Highest quintile of depressive symptoms	0.17	
ADLs	0.20	(0.75)
IADLs	1.08	(1.99)
Walking speed (m/s)	0.39	(0.17)
Grip strength	26.50	(9.69)
Observations	5059	

Table 1 presents descriptive statistics for our sample at baseline. The average age of respondents in the baseline wave was 62.39 years and a slight majority was female. The sample consisted of individuals with low education - understandable given the inferior education provided to Africans under apartheid - as approximately 46 % had no formal education and 34 % had some primary education only. In terms of marital status, most individuals in the sample were either married/in partnership (51 %) or widowed (30 %). Approximately 70 % of the sample was born in South Africa. The remaining 30 % of were mostly born in Mozambique and emigrated to South Africa during the Mozambican civil war. Despite being born outside of South Africa these individuals are eligible for the old age state grant. In line with persistently high unemployment rates, only 16 % of individuals reported they were working at baseline. In our sample, average monthly consumption per capita was approximately ZAR 1,584 Rands which was roughly 100 USD by the end of 2015.

At baseline approximately 50 % of the sample received the old age state grant and on average these individuals had been eligible for 5.82 years. To provide further details, we find that at baseline 44 % of individuals had not been eligible for the old age grant, while 14 % had been eligible for 1–5 years, 16 % from 6 to 10 years and 25 % had been eligible for more than 10 years. In terms of health outcomes, 68 % of individuals reported being in good or very good health at baseline. The average number of limitations in activities of daily living (ADLs) and instrumental activities of daily living (IADLs) was 0.2 and 1.08. Finally, the average gait speed and grip strength were 0.39 m/s and 26.50 kg/m2 respectively. Appendix Table 2 compares the descriptive statistics between our final sample and those individuals who are not followed up in the second wave. In line with other ageing surveys, we find that individuals in our sample are younger and healthier at baseline. However, once we control for age, individuals not available in the second have less exposure to the old age state grant.

**Table 2:** Association Between Old Age Grant Exposure and Three Composite Health Indices.

	(1)	(2)	(3)
	Health status index	Functionality index	PVW Health status index
**Panel A**			
Log[Exposure to pensions + 1]	0.058*	0.086^***^	0.062^***^
	(0.023)	(0.023)	(0.018)

**Panel B**			
Pension exposure: 1–5 years	0.114^**^	0.109^***^	0.087^**^
	(0.035)	(0.031)	(0.028)
Pension exposure: 6–10 years	0.147^**^	0.137^**^	0.098^**^
	(0.045)	(0.044)	(0.035)
Pension exposure: More than 10	0.123	0.182*	0.070
	(0.068)	(0.071)	(0.053)
Observations	9,192	9,192	9,192

Estimates from OLS regressions controlling for gender specific quadratic age functions, marital status, birthplace and education controls. Individual clustered standard errors in parenthesis. All three indices- *health status, functionality, and PVW health status-* were constructed such that higher values imply better health.

* *p* < 0.05, ^**^p < 0.01, ^***^p < 0.001.

### Empirical approach

To evaluate the relationship between the potential exposure to the old age state pension and health, we exploit the changes in age eligibility over time and estimate the following main equation:

$$Y_{\mathrm{it}}=\alpha_{i}+\beta_{1}*\log\left({\mathrm{Exposure}}_{\mathrm{it}}+1\right)+f\left({\mathrm{Age}}_{\mathrm{it}},{\mathrm{Gender}}_{\mathrm{it}}\right)+\gamma X_{i}+\delta_{W}+\varepsilon_{\mathrm{it}}$$

Where $Y_{\mathrm{it}}$ are our health outcomes, $\alpha_{i}$ is an individual–level random intercept, ${\mathrm{Exposure}}_{\mathrm{it}}$ is the number of years individual *i* has been potentially exposed to the old age pension grant at time *t*, $f\left({\mathrm{Age}}_{\mathrm{it}},{\mathrm{Gender}}_{\mathrm{it}}\right)$ is a gender–specific age–polynomial, $X_{i}$ is a vector of controlling variables, $\delta_{W}$ are the HAALSI wave fixed effects, and $\varepsilon_{\mathrm{it}}$ is the idioscrynatic error term.

In terms of the outcomes our main results evaluate the effect of pension exposure on health using the three health indices described before: *health status, disability status* and *PVW health status*. In addition, we conduct a series of secondary analyses to evaluate the role of the different outcomes, and therefore used as outcomes a set of self–reported health measures which consist of a binary indicator for reporting to be in good or very good self–reported health (SRH), a standardised measure of the number of depressive symptoms, the number of limitations in activities of daily living (ADLs), the number of limitations of instrumental activities of daily living (IADLs), walking speed, and grip strength.

In our main models, we evaluate the role of pension exposure in two ways. In the first we use the cumulative impact of being exposed to the old age pension grant. This is estimated through the measure of the number of years exposed. In our models we use the log transformation of the number of exposed years to account for the non–linear relation existent between pension exposure and health. In a second set of analyses, we define categories of pension exposure based on years and include three categories of exposure: 1–5 years, 6–10 years, and more than 10 years. These categories were defined such that individuals are similarly distributed across all categories and to serve as confirmation for the results in the main models.

In this study we used eligibility rather than actual take-up of pension for two reasons. First, in South Africa a high proportion of individuals take up the old age grant once they are eligible. Data from HAALSI show that over 85 % of individuals for the old age grant are receiving it. Therefore, there is little difference between potential and actual take up, but there is no information on when these individuals started receiving the old age grant. Second, the actual take–up of the old age grant depends on individual behaviours and is therefore endogenous. By using the potential take-up, we exploit only the timing of birth and the pension reforms thus estimating the reduced form impact of old age grant on health. Use of pension eligibility implies that we estimate the Intent–to–treat (ITT) effect of the old age grant on health. Since this is an underestimate of the actual effects of receiving the old age grant, our estimates are likely to represent a lower bound of the health effect of the actual receipt of the old age state grant.

In all models we include a gender–specific polynomial age trend to account for potential non–linear associations between age and health, also to account for differences in trends that exist across gender. For our main results, we use a gender–specific second order polynomial that represented the best fit in the data. However, we present in the appendix results testing the robustness of our findings to a first order polynomial trend. Furthermore, the vector of controlling variables includes marital status, education level, and whether the individual was born in South Africa. Analysis using the general South African population would also require including race as a determinant of pension eligibility, however all the population covered by HAALSI is African and therefore there are no race differences that determine eligibility to the old age pension grant in our sample. Finally, our models include an individual random intercept and survey wave fixed effects. Across all models we obtain individual clustered robust standard errors.

## Results

The results concerning the association between pension exposure and the three health indices are presented in Table 2. Panel A, specifically, explores the association of the direct and cumulative effect of pensions on three composite health indices. In the case of the *health status* index, we find that being exposed to the old age pension grant is associated with better general self–reported health. We also find that a 10 % increase in the number of years of exposure to the old age grant is associated with a 0.005 SD better *health status* (β=0.057; p-value = 0.013; 95 % CI: 0.012–0.102). The results for the *functionality index* portray a similar pattern. In this case, being exposed 10 % more years to the old age grant is associated with a 0.008 SD higher self–reported functioning (β=0.086; p–value < 0.001; 95 % CI: 0.041–0.132). Finally, in the third column, we find similar effects for the *PVW Health Status* index. That is, being exposed to the old age grant is positively associated with better health with a 10 % increase in the number of years exposed to the old age grant leading to a 0.006 SD reported better health (β=0.062; p–value = 0.001; 95 % CI: 0.026–0.097). These results suggest that pension exposure has a positive cumulative effect on health.

Panel B in Table 2 presents the results for the models where the number of years exposed to the old age pension grant program is grouped into categories. Across all results we find that being exposed to the old age grant is likely beneficial to an individual’s health. In column 1, we find that the coefficients for the three categories of pension exposure are positively associated with a higher *health status* index. Being exposed between 1 and 5 years is associated with a 0.114 SD higher *health status* index (p–value = 0.001; 95 % CI: 0.045–0.183). Similarly, being exposed between 6 and 10 years is associated with better health (β=0.147; p–value-=0.001; 95 % CI: 0.059–0.235). Finally, being exposed for more than 10 years is also associated with a 0.123 SD higher *health status* index (p–value = 0.069; 95 % CI: −0.009–0.256).

Column 2 shows a similar pattern of results for the *functionality index*. We find that being exposed to the old age grant is associated with better health. Specifically, being exposed between 1 and 5 years, being exposed 6 to 10 years, and being exposed more than 10 year leads to a 0.109 (p–value < 0.001; 95 % CI: 0.048–0.170), 0.37 (p–value = 0.002; 95 % CI: 0.051–0.223), and 0.182 (p–value = 0.010; 95 % CI: 0.043–0.322) SD higher *functionality index* respectively. Finally, column 3 presents the results for the *PWV Health status* index and leads to similar conclusions. All pension exposures are positively associated with better health. In the first category, being exposed between 1 and 5 years, leads to a 0.087 SD higher health status index (p–value = 0.002; 95 % CI: 0.033–0.141). Being exposed between 6 and 10 years, leads to 0.098 SD better health status (p–value = 0.006; 95 % CI: 0.029–0.167); and finally, more than 10 years of pension exposure led to 0.070 SD higher health status (p–value = 0.187; 95 % CI: −0.034–0.174). Appendix Table 3 shows that we reach the similar conclusions when using linear gender–specific age trends in our models.

**Table 3:** Association Between Old Age Grant Exposure and Components of the Health Indices.

	(1)	(2)	(3)	(4)	(5)	(6)
	SRH	Depression score	ADLs	IADLs	Walking speed	Grip strength
**Panel A**						
Log[Exposure to pensions + 1]	−0.000	0.000	−0.061^***^	−0.145*	−0.003	0.024
	(0.011)	(0.009)	(0.018)	(0.060)	(0.015)	(0.311)

**Panel B**						
Pension exposure: 1–5 years	0.010	0.001	−0.071^**^	−0.028	−0.018	0.926
	(0.020)	(0.016)	(0.026)	(0.103)	(0.026)	(0.550)
Pension exposure: 6–10 years	0.005	0.013	−0.076*	−0.223	0.003	0.906
	(0.023)	(0.019)	(0.035)	(0.118)	(0.031)	(0.649)
Pension exposure: More than 10	−0.017	−0.007	−0.142*	−0.471^**^	−0.030	0.639
	(0.031)	(0.025)	(0.056)	(0.178)	(0.039)	(0.828)
Observations	9,179	9,068	9,141	3,653	4,151	3,315

Estimate from OLS regressions controlling for gender specific quadratic age functions, marital status, birthplace and education controls. Individual clustered standard errors in parenthesis. Each column corresponds to a different outcome. Column 1 presents the effects for good or very good self–reported health. Column 2 presents the results for the number of depressive symptoms where a higher number implies worse mental health. Column 3 presents the results for the number of limitations in activities of daily living. Column 4 presents the results for the number of limitations in instrumental activities of daily living. In both cases, a higher number implies more limitations and therefore worse health. Column 5 presents the walking speed with higher values being representative of better health. Column 6 presents the results for grip strength where higher values represent better health.

* *p* < 0.05, ^**^p < 0.01, ^***^p < 0.001.

Overall, the results from Table 2 show that pension exposure is likely to be associated with better health. However, these results do not allow us to understand what aspects of the index are being affected by pension exposure. To explore this in further detail we present in Table 3 the association between pension exposure and selected components of the health indices used above.

In contrast to the main results on the composite indices, there is greater variation in the estimates for specific components of the indices. Nevertheless, we find that pension exposure is mostly associated with better self-reports of ADLs and IADLs. From Panel A, we see that cumulative exposure to the old age grant is associated with an improvement in the individual performance of ADLs. More specifically, a 10 % increase in exposure to the old age grant reduces by 0.006 (β=-0.061; p-value = 0.001; 95 % CI: −0.097 - −0.252) the number of limitations in activities of daily living reported. This finding is reinforced by the results concerning ADLs in Panel B. There, we see that each category of pension exposure is also associated with a reduction in the reporting of limitations in activities of daily living, and more importantly, that greater exposure is associated with an even lower number of limitations. We find that being exposed between 1 and 5 years leads to an improvement in the number of ADLs by 0.072 (p–value = 0.007; 95 % CI: −0.123 - −0.020), being exposed between 6 and 10 years leads to a reduction in ADLs of 0.076 (p–value = 0.029; 95 % CI: −0.145 - −0.008) and being exposed more than 10 years to the old age grant reduces ADLs by 0.142 (p–value = 0.011; 95 % CI: −0.252 - −0.033) on average.

The results for IADLs lead to similar conclusions. From Table 3 Panel A we can see that the cumulative exposure to the old age grant leads to a reduction in the number of limitations. That is, we find that a 10 % increase in the number of years eligible for the old age grant leads to a reduction of 0.014 limitations of instrumental activities of daily living reported (β=-0.145; p–value = 0.016 95 % CI: −0.263 - −0.267). In the case of IADLs, we also can see in Panel B, that as the category of exposure increases there is a greater improvement in the performance of instrumental activities of daily living. We find that being eligible between 6 and 10 years for the old age grant leads to a reduction in the IADLs of approximately 0.223 units (p–value = 0.060; 95 % CI: −0.455 – 0.010). Similarly, being exposed for more than 10 years is associated with a 0.471 lower number of IADLs (p–value = 0.008; 95 % CI: −0.819 - −0.123). In the remaining columns of Table 3 we find no link between pension exposure and good self–reported health, the number of depressive symptoms- mental health-, walking speed or grip strength in our main results. The findings using gender–specific age trends (Appendix Table 4) lead to similar conclusions but showcase even stronger associations between pension exposure and health.

## Discussion and conclusion

The objective of this study was to evaluate the association between pension exposure and dimensions of health amongst older persons in South Africa. For this we used longitudinal data from the HAALSI study in northeast South Africa (Agincourt) which has generated rich economic and demographic information. Overall, we show that the accumulated exposure to pensions is positively associated with measures of better health. To obtain a more nuanced understanding of the association between pension exposure and health, we conducted similar analyses on some of the main items used to construct the index. These findings affirm our main results and show that pension exposure is likely to positively influence self–reported disabilities. However, cumulative eligibility over time to social grants was not associated with self–reported measures linked to general or mental health.

The effects we find of pension exposure on reported disabilities are possibly explained by previous research from South Africa showing that income from old age grants is often used to support food consumption (Armando Barrientos, 2003; Marianne Bertrand et al., 2003; Case and Deaton, 1998; Esther Duflo, 2003, Lloyd-Sherlock and Agrawal, 2014). Current studies show that an appropriate diet at older ages can offset sarcopenia – loss of muscle mass - which in turn will influence the physical capacity of individuals (Sian M Robinson, 2018; Xu et al., 2020). In the case of South Africa, the old age grant may offer the possibility for household to obtain better food or to invest in health maintaining products (Peter Lloyd-Sherlock and Sutapa Agrawal, 2014) which may then in turn contribute to maintaining a better physical condition amongst. Nevertheless, research on the mechanisms between pension exposure and health is scarce in low and middle–income countries.

Our results provide supporting evidence to other findings currently in the literature. In the same South African setting, another study evaluating the ITT effect of pensions on health found that the wellbeing of women only improved in the early years following pension–eligibility thus suggesting the presence of a positive but transitory effect (Enid Schatz, Xavier Gómez-Olivé, Margaret Ralston, Jane Menken and Stephen Tollman, 2012). Our results confirm the existence of an effect but drawing on a wider set of health indicators suggest that such an association is sustained rather than temporary. Other South African studies explored associations between residence in an old age grant recipient household and an association with self–reported health, management of hypertension or quality of life but found no effect (Lloyd-Sherlock and Agrawal, 2014, Lloyd-Sherlock et al., 2020). In our study we obtain similar findings, with pension exposure not linked with self–reported general health or mental health but do find an effect of pension exposure on health indices and indicators related to physical disabilities. The similarity in null findings coupled with contrasts in findings suggests that the effect of the old age grant may not take place in all domains of health. We find that pension exposure is primarily associated with measures relating to the domain of self–reported disabilities. This highlights that old age social grants in South Africa are potentially beneficial in either delaying the onset of physical disabilities or helping cope with them.

We also contribute to the general literature on old age pensions and health across low and middle–income countries. As in South Africa, our findings reinforce the potential link between old age social protection schemes and healthy ageing. In Mexico, recent studies evaluating the effects of a non–contributory pension program found that receipt of pension income led to improvement in health-related biomarkers, although in this case the improvements were most likely due to increased expenditures in health care (Aguila et al., 2015, Águila et al., 2018, Riumallo-Herl and Aguila, 2019). Another study evaluating the role of social pensions in a wider set of countries- including South Africa- found that old age pensions could lead to health improvements, but that such improvements were contingent on the level of education attained (Peter Lloyd‐Sherlock, Nadia Minicuci, John Beard and Somnath Chatterji, 2012). Our paper thus contributes to a growing literature on the potential beneficial impacts of social pensions on health.

Examining the literature indicates our study has several advantages. The first is that we used data from a comprehensive ageing survey which provided us with a wider array of health domains to evaluate. Availability of this data allowed us to construct health indices that compare to studies conducted in high-income countries and gain a comprehensive perspective on how social pensions can influence health. A second advantage is that we explore the association with eligibility to the old age pension which is exogenous and only determined by the date of birth and interview. This however leads to a limitation of our study, namely the fact that we only estimate the 'intention to treat' (ITT) effects and not the direct effect of receiving the pension. This however is not a major limitation since the ITTs are likely to represent the lower bound of the effect of pensions on health in South Africa. Another limitation in this study is attrition between waves. We find however that conditional on age, individuals lost to follow have less exposure to the old age state grant. This further suggests that we underestimate the effect of pension exposure on health. A second limitation is that our measure of exposure does not represent the amount of time an individual has received the old age state grant. However, since most individual’s uptake the pension in the years after becoming eligible these values are strongly correlated. Furthermore, old age grant uptake rates are above 80 % thus suggesting only a small minority of individuals do not take up the grants (Margaret Ralston, 2016, Schatz, 2012). A final limitation is our choice of outcomes that has been guided by previous economics of ageing literature in surveillance sites. Within each dimension we have found that pension exposure is only associated with physical functioning. This could imply that pension exposure might also not be associated with other markers of health.

Our findings suggesting that the non-contributory old age pension grant provided to eligible rural-dwelling South Africans is associated with better health provide further argument to implement and expand such programmes in other middle and low-income countries. As populations continue to age, reflected in rising life expectancy, policy makers need to consider the different tools and mechanisms at their disposal not only to protect the elderly financially but also to encourage healthy ageing and ultimately a 'compression of morbidity'. The results presented contribute to that policy discussion by highlighting how old age pensions can lead to healthier ageing while postponing the onset of physical limitations.

## CRediT authorship contribution statement

**Carlos Riumallo Herl:** Conceptualization, Methodology, Formal analysis, Writing – original draft. **Chodziwadziwa Kabudula:** Methodology, Data curation. **Kathleen Kahn:** Conceptualization, Writing – review & editing. **Stephen Tollman:** Conceptualization, Writing – review & editing. **David Canning:** Conceptualization, Methodology, Writing – review & editing, Supervision.

## Declaration of Competing Interest

The authors declare the following financial interests/personal relationships which may be considered as potential competing interests: Carlos RiumalloHerl, Chodziwadziwa Kabudula, Kathleen Kahn, Stephen Tollman, and David Canning report no conflicts of interest

## Figures and Tables

**Fig. A1:**
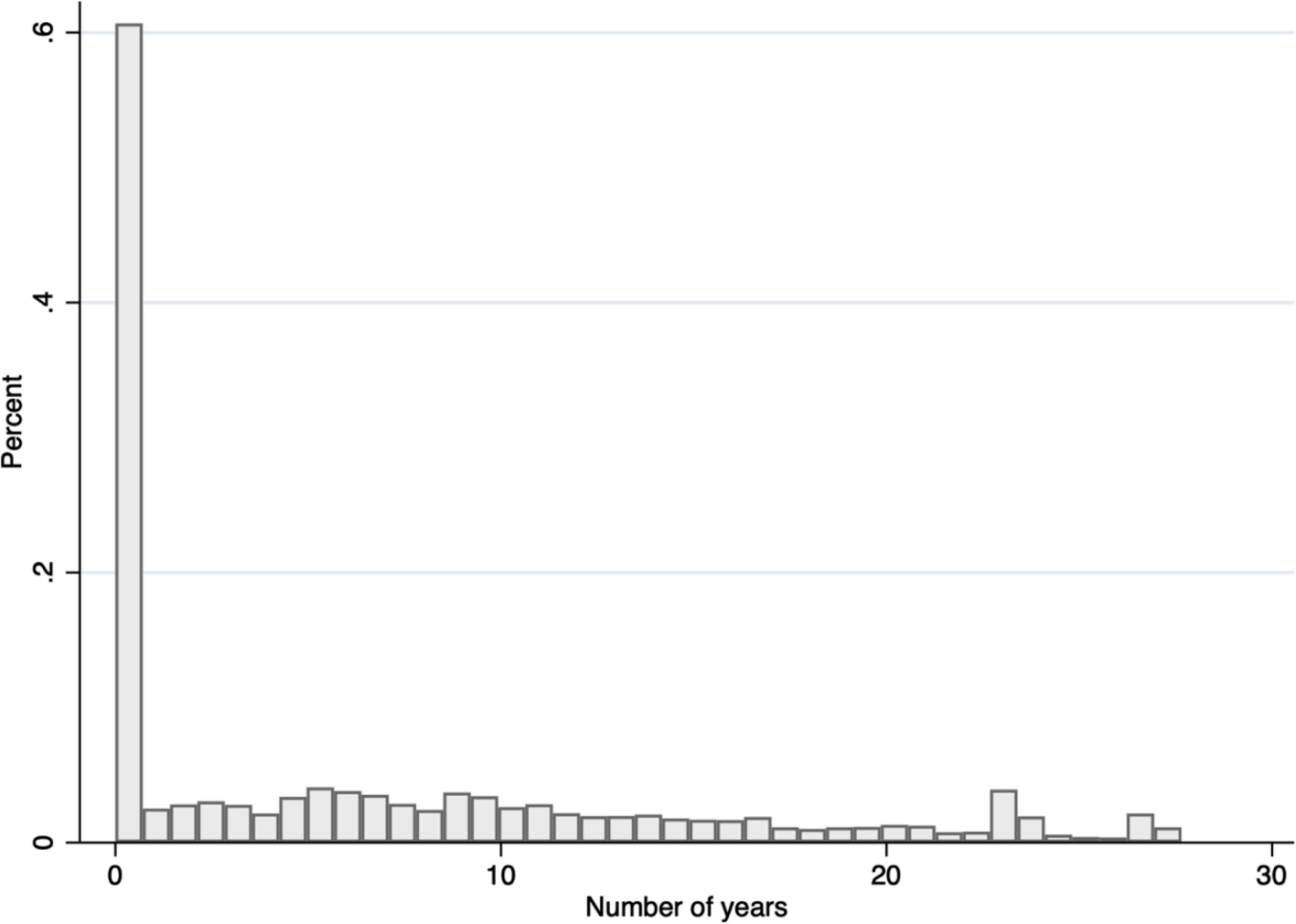
Distribution of old age state grant eligiblity years.

**Fig. A2:**
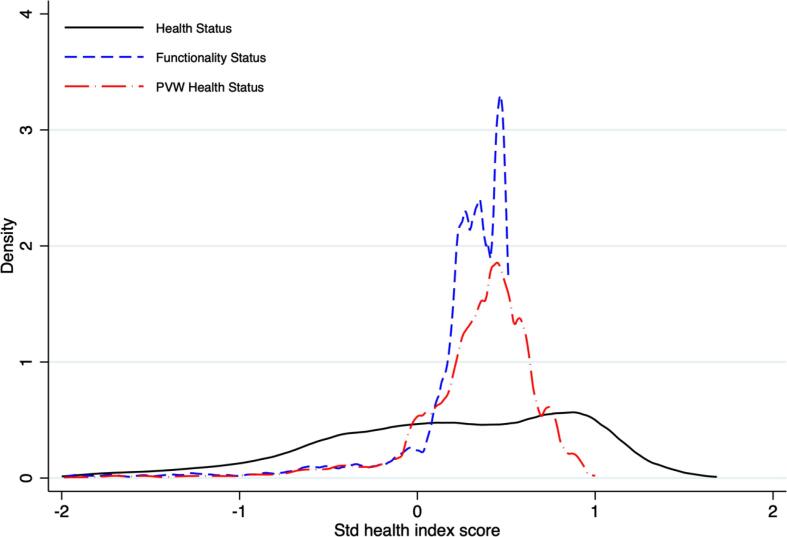
Distribution of health indices.

**Table A1:** Variables included in each of the health indices and their scoring coefficients.

Items	Health Status	Disability Status	PVW	
*Mobility*				
No difficulty crossing a room	0.034	0.373	0.361	
Completed normal walk	0.365	0.259	0.251	
Completed semi tandem	0.393	0.268	0.265	
*Self-care*				
No difficulties dressing	0.390	0.381	0.362	
No difficulties bathing	0.395	0.393	0.374	
No difficulties eating		0.260	0.246	
No difficulties getting out of bed		0.398	0.380	
No difficulties using toilet		0.397	0.381	
*Health*				
Categorical self-reported health (1 Poor-5 Excellent)			0.188	
No back problems			0.021	
No heart problems			0.031	
Never suffered stroke			0.151	
Does not suffer from hypertension (measured and reported)			0.053	
No respiratory problems			0.026	
Does not suffer from diabetes (measured and reported)			0.079	
Normal BMI			0.052	
*Health care use*				
No hospital stays in last 12 months			0.079	
No doctor visits in last 3 months			0.066	
*Pain and discomfort*				
No reported physical pain yesterday	0.241			
*Cognition*				
No difficulties concentrating	0.298	0.109	0.103	
No difficulties learning new things	0.277	0.091	0.085	
*Sleep/Energy*				
Never had difficulties sleeping in past 4 weeks	0.187			
*Affect*				
Never felt sad or depressed in last two weeks	0.284	0.136		
*Vision*				
No reported visual difficulties	0.254			
*Work*				
Health does not limit work			0.121	

**Table A2:** Attrition analysis.

	Sample	Attritors: Difference	Attritors: P-val
Age	63.265	5.463	<0.001
Male	0.446	0.102	<0.001
No formal education	0.445	0.070	<0.001
Some primary education	0.345	−0.029	0.092
Some secondary education	0.119	−0.031	0.004
Secondary or higher education	0.090	−0.009	0.381
Never married	0.067	0.013	0.160
Married or in partnership	0.484	−0.057	0.002
Separated or divorced	0.126	0.005	0.694
Widowed	0.323	0.039	0.026
Born in South Africa	0.694	0.024	0.159
Working	0.163	−0.035	0.006
Total household consumption per capita	1633.452	−163.742	0.017
Old age pension	0.546	0.109	<0.001
Number of years eligible for pension	6.375	2.951	<0.001
Health status index	0.048	−0.534	<0.001
Functionality status index	0.035	−0.505	<0.001
PVW Health status index	0.267	−0.523	<0.001
Good or very good self-reported health	0.649	−0.147	<0.001
Highest quintile of depressive symptoms	0.178	0.062	<0.001
ADLs	0.189	0.305	<0.001
IADLs	1.084		
Walking speed (m/s)	0.439	−0.075	<0.001
Grip strength	26.856	−1.583	<0.001

Note: Column 1 presents the sample mean at baseline. Columns 2 and 3 present the coefficient and p-value estimate of the difference between those in the sample and attritors. Many health difference are due to age, once age is controlled for attritors have been exposed less to the old age state grant.

**Table A3:** Association between old age grant exposure and health indices.

	(1)	(2)	(3)
	Health status index	Disability index	PVW Health status index
**Panel A**			
Log[Exposure to pensions + 1]	0.101^***^	0.106^***^	0.078^***^
	(0.025)	(0.025)	(0.020)

**Panel B**			
Pension exposure: 1–5 years	0.262^***^	0.273^***^	0.111^***^
	(0.039)	(0.038)	(0.030)
Pension exposure: 6–10 years	0.346^***^	0.329^***^	0.146^***^
	(0.053)	(0.056)	(0.040)
Pension exposure: More than 10	0.226^**^	0.230^**^	0.136*
	(0.074)	(0.078)	(0.057)
Observations	8,143	8,143	8,143

Estimate OLS regressions controlling for gender specific linear age functions, marital status, birthplace, and education controls. Individual clustered standard errors in parenthesis. All three indices- *health status, functionality, and PVW health status-* were constructed such that higher values imply better health.

* *p* < 0.05, ^**^p < 0.01, ^***^p < 0.001.

**Table A4:** Association Between old Age Grant Exposure and Health Measures.

	(1)	(2)	(3)	(4)	(5)	(6)
	SRH	Depression score	ADLs	IADLs	Walking speed	Grip strength
**Panel A**						
Log[Exposure to pensions + 1]	0.009	−0.005	−0.068^***^	−0.336^***^	0.014	0.110
	(0.012)	(0.010)	(0.020)	(0.072)	(0.015)	(0.321)

**Panel B**						
Pension exposure: 1–5 years	0.040*	−0.012	−0.179^***^	−0.670^***^	0.028	0.801
	(0.020)	(0.016)	(0.031)	(0.115)	(0.025)	(0.523)
Pension exposure: 6–10 years	0.041	−0.010	−0.194^***^	−0.966^***^	0.055*	1.023
	(0.024)	(0.020)	(0.045)	(0.148)	(0.028)	(0.605)
Pension exposure: More than 10	−0.003	−0.021	−0.157*	−0.962^***^	0.019	0.464
	(0.033)	(0.027)	(0.062)	(0.215)	(0.039)	(0.852)
Observations	8,140	8,034	8,101	3,196	3,634	2,934

Estimate OLS regressions controlling for gender specific linear age functions, marital status, birthplace, and education controls. Individual clustered standard errors in parenthesis. Each column corresponds to a different outcome. Column 1 presents the effects for good or very good self–reported health. Column 2 presents the results for the number of depressive symptoms where a higher number implies worse mental health. Column 3 presents the results for the number of limitations in activities of daily living. Column 4 presents the results for the number of limitations in instrumental activities of daily living. In both cases, a higher number implies more limitations and therefore worse health. Column 5 presents the walking speed with higher values being representative of better health. Column 6 presents the results for grip strength where higher values represent better health.

* *p* < 0.05, ^**^p < 0.01, ^***^p < 0.001.

## Data Availability

Data is publicly available in Harvard Dataverse (https://dataverse.harvard.edu/)
